# Resource, Collaborator, or Individual Cow? Applying Q Methodology to Investigate Austrian Farmers' Viewpoints on Motivational Aspects of Improving Animal Welfare

**DOI:** 10.3389/fvets.2020.607925

**Published:** 2021-01-12

**Authors:** Lorenz Maurer, Josef Schenkenfelder, Christoph Winckler

**Affiliations:** Division of Livestock Sciences, Department of Sustainable Agricultural Systems, University of Natural Resources and Life Sciences, Vienna, Austria

**Keywords:** dairy, cow, welfare, improvement, farmer, viewpoint, motivation, Q methodology

## Abstract

One keystone to successful welfare improvement endeavors is a respected cooperation between farmer and advisor (e.g., veterinarian), which requires a thorough understanding of what motivates farmer behavior. In this respect, Q methodology offers a promising approach in investigating individual motivational patterns and to discriminate between and describe typologies of farmers. In our study we explored, based on a sample of 34 Austrian dairy farmers, how 39 potentially motivating statements regarding the improvement of dairy cow health and welfare were assessed. We were able to identify and describe four different viewpoints, explaining 47% of total study variance. All four viewpoints have in common that pride in a healthy herd is motivating to work toward improved animal health and welfare to a certain extent, but meeting legal requirements is rather not. Viewpoint 1 acknowledges welfare for economic performance, ease of work and short working hours but does not make allowance for outside interference. Participants loading on Viewpoint 2 also show a focus on economic aspects but, keep close track of the animal welfare debate recognizing its potential to improve the public image of dairy farming. Even though they cautiously criticize an exploitative application of dairy farming, they do not want to be understood as role models. With regards to animal welfare, farmers sharing Viewpoint 3 perceive themselves as superior to and show little reluctance of comparison with mainstream farming. For them, the animal as sentient being itself owns some intrinsic value and it is necessary to strike a balance between economic and other, ethical considerations. Viewpoint 4 perceives cows as equal collaborators who deserve to be treated with respect and appreciation and is willing to accept certain economic losses in order to maintain high standards regarding animal health and welfare. Using Q methodology, we have been able to draw high resolution images of different farmer typologies, enabling advisors to tailor intervention strategies specifically addressing leverage points with a high chance of farmer compliance.

## Introduction

Despite extensive research over the past decades and a wealth of knowledge about risk factors [e.g., regarding claw health and lameness (1–4), mastitis (5–7), cleanliness (8, 9), integument alterations (9–11) or mortality (12, 13)], ensuring good animal health and welfare (AHW) in dairy herds remains a difficult task. The prevalence of production diseases such as lameness and mastitis has changed rather little since the first introduction of herd health programs during the 1970s (14–17). AHW planning as a novel form of cooperation between farmer and advisor (e.g., veterinarian) has been proposed as one approach in addressing such persisting problems (18). Identification of farm-specific challenges and individual farmer goals and the joint development of action plans may benefit the animals to some degree (19), as it has been shown that farmers can differ in what motivates them and in the relative importance they attribute to different benefits from improved AHW (20). However, farmer behavior (e.g., compliance with action plans) is decisive for the success or failure of such programs (17, 21). Advisors, therefore, should be able to support farmers by facilitating behavioral change in the way of a consultant or coach, rather than a provider of knowledge or single cow therapist only (22–24). In this regard, it is crucial to develop a thorough understanding of what motivates farmer behavior in the context of improving AHW and of how farmers may differ in their motivation.

Good AHW may have a range of benefits for farmers (20). Zoo-technical performance impacts on financial revenue and economic sustainability (e.g., milk yield, costs of illness), but work satisfaction as well as feelings of responsibility or empathy toward animals as sentient beings may also be important motivators to strive for better AHW (25). According to McInerney (26), the value of animal health and welfare is a subjective weighting put on it by an individual (e.g., the farmer), reflecting the perceived benefit or cost associated with it. McInerney distinguishes between “use values” and “non-use values” attributable to the well-being of farmed animals. As a resource in livestock farming, animals contribute to the production process. This “use value” by itself warrants a certain amount of care to be given to the animals' well-being (e.g., feeding, housing, veterinary attention), but only inasmuch as it is necessary for achieving the desired use of the animal within the production process. Much effort has gone into calculating the direct and indirect costs of diseases such as lameness and mastitis as well as the costs and cost-efficiency of control measures (27, 28), hoping that better knowledge and raised awareness will lead to improved AHW (29). Still, other research suggests that the provision of knowledge only (22) or the promotion of financial benefits from improved welfare (3, 30) are often not sufficient to initiate change in stockpersons' behavior, indicating that not only financial or production-related considerations are involved in decision making regarding AHW. There may be other tangible benefits of good AHW, such as the farmer having more time for other things and an improved work environment. These in turn may lead to intangible benefits such as increased job satisfaction and have been shown to be very important to farmers (20, 25).

While use-related values are arguably important in the context of animal husbandry, it also is immediately apparent that farmers may attribute value to the well-being of livestock independently from any use in the production process. These “non-use values” focus on positive feelings that arise from knowing that animals are being treated appropriately (31) or, conversely, from avoiding feelings of unease or discomfort that arise when an individual perceives that sentient beings suffer in their role as resource (26). Similarly, a feeling of pride in a healthy herd seems to be an important driver for farmers to ensure good welfare (3). The concept of subjective norms, which forms part of Ajzen's Theory of Planned Behavior (TPB) (32), tries to explain whether a feeling of pride is motivated by intrinsic or extrinsic factors, i.e., the desired impression on or recognition by reference groups such as fellow farmers, veterinarians, or a dairy company (20, 33). The TPB is probably the dominant theoretical framework for studying farmer attitude and behavior with regards to AHW (15, 33–38). However, the model's conceptual design to understand and predict human behavior works best for very particular questions (e.g., intention to improve the management of foot lesions) but is less suited to explore intentions to change behavior on a more general level (e.g., intention to improve animal welfare on the farm) (21). Furthermore, the TPB follows a rationalistic approach, which might miss relevant motives explaining farmer behavior regarding animal health and welfare.

Considering the wide range of perceived benefits and their interdependence affecting farmer behavior as well as potential differences between individuals in what motivates them, a comprehensive understanding of farmer motivation is necessary when trying to stimulate behavioral change in farmers in order to improve AHW (39, 40). Advisory strategies building on such an understanding and taking individual farmer motivation into account, promise to be more effective than one-size-fits-all approaches (20, 38). Yet, few attempts have been made to investigate individual differences between farmers, while this has been suggested for future research to further the understanding of farmer motivation regarding AHW (31).

Standard approaches to understand attitudes include qualitative (e.g., focus group discussions, interviews) and quantitative (e.g., questionnaire) techniques. The first, however, is often criticized for lacking statistical rigor and the latter for being prone to bias based on misunderstood questions or categories (41). In this study, we applied Q methodology to explore shared viewpoints among a sample of Austrian dairy farmers asking what they perceive as motivating to ensure good health and welfare of their livestock. Q methodology was first introduced in 1935 by William Stephenson (42) and is suited for exploratory and theory-generating research, aimed at revealing shared subjective viewpoints and creating taxonomies of these viewpoints (43). Contrary to conventional by-item factor analysis (a so-called R methodological technique), it incorporates a by-person factor analysis, which combines quantitative and qualitative aspects of social research (44) in order to group similar patterns of thought on a specific topic of interest (45). This means that in a Q study participants are considered as variables and test items as population, thus essentially inverting the R methodological tradition. It allows to develop a holistic image of individuals and to thoroughly contrast their differences based on rigorous statistical methods. To this end it is not possible to merely transpose data gathered for R methodological purposes but it requires new data where items are measured relatively to one another by a collection of participants (41, 46). The goal of a Q methodological study is not to estimate what proportion of the population shares certain viewpoints (47). Rather, it is concerned with establishing the existence of certain shared viewpoints and with understanding and comparing them (46). The method has been applied in a wide range of disciplines such as political science (47, 48) and health psychology (44, 49), but also in the fields of agricultural and veterinary sciences (50–54). For example, Q methodology has been used to examine differences in management styles among farmers (54) and to better understand farmers' viewpoints, e.g., dairy farmers' expectations related to their participation in a herd health management program (51).

With our study we intend to add to a comprehensive picture of farmers' viewpoints toward working with AHW, investigating (i) how a set of potentially motivating statements regarding the improvement of dairy cow health and welfare is assessed by farmers and (ii), whether, based on this assessment, groups of respondents sharing the same viewpoint can be identified, characterized and contrasted. Such an understanding may help to develop appropriate communication approaches and successful advisory services.

## Farms, Materials, and Methods

### Selection of Participants

The group of participants, the so-called P set, was selected from among 2,600 family-run dairy operations of an Austrian dairy company. Sampling criteria listed in Table 1 were determined by availability of information on farm characteristics, as well as expected associations of these characteristics with distinct viewpoints on improving animal welfare. The goal of the P set design was to allow a wide range of viewpoints to be expressed by study participants and was therefore based on the abovementioned theoretical considerations. In a first step, 73 eligible participants were contacted by representatives of the dairy company and asked for consent to take part in the study. Those who agreed were subsequently contacted by LM to explain the aim of the study and to arrange an appointment for data collection. LM visited all participants between December 2018 and February 2019 at their homes, where the sorting procedure was performed, the post-sorting interview conducted and additional demographic data (see Table 1) was collected. The selection and acquisition process resulted in a final P set of 35 farmers, who did not receive any compensation for their participation. It should be noted, that in Q methodological studies, the validity of the statistical inferences involved in establishing the existence of a factor does not depend on large numbers of participants (47). In contrast, suggestions for the minimum number of participants in by-item factor analysis based on a conventional questionnaire-survey approach utilizing scale items (R methodological techniques) vary roughly between 100 and 500, depending on particular study conditions (55).

**Table 1:** Descriptive characteristics of all participants (P set) and of the participants allocated to extracted factors F1–F4.

	**P set (*n* = 34)**		**F1 (*n* = 8)**	**F2 (*n =* 5)**	**F3 (*n =* 5)**	**F4 (*n =* 3)**
Age (median; min/max)	46; 31/65		48; 31/56	50; 36/54	44; 32/65	44; 32/57
Gender (female/male)	11/23		3/5	1/4	0/5	1/2
Contribution of dairy farming in % to household income (min/max)	25/100		50/100	50/100	25/100	25/50
**Sampling criteria**	**%**	***n***	***n***	***n***	***n***	***n***
Operation type^*^						
Conventional (GMO-free)	38	13	4	2	1	
Organic	62	21	4	3	4	3
**Feeding regime**^†^						
GMO-free “standard”	23	8	2	1		
GMO-free “haymilk”	15	5	2	1	1	
Organic “standard”	29	10	3		2	1
Organic “haymilk”	15	5	1	1	1	1
Organic “Goldstandard”	9	3		1	1	
Organic “Reine Lungau”	9	3		1		1
**Access to pasture**						
Yes	79	27	5	4	5	3
No	21	7	3	1		
**Housing system**						
Tie-stall (all year)	9	3	1			
Tie-stall (with access to outdoor run and/or pasture)	32	11	4	2	1	
Loose housing	59	20	3	3	4	3
**Herd size**^‡^						
Q1	15	5	1			2
Q2	18	6	3		1	
Q3	38	13	1	4	3	1
Q4	29	10	3	1	1	

* *Conventional milk is produced GMO-free according to the Austrian Food Codex. Organic milk is produced according to EU organic regulations*.

† *Haymilk is a quality scheme protected under the label “Traditional speciality guaranteed” by the European Commission. Essentially, the haymilk regulatory prohibits feeding fermented feed (i.e., silage, but also certain by-products of the food industry). Haymilk may be produced conventional or according to EU organic regulations. For details see: https://bit.ly/2M0OeEU (accessed on September 8, 2020). Organic “Goldstandard” and Organic “Reine Lungau” represent different Austrian private label schemes with restrictions to mainly feeding, housing and access to pasture, and/or an outdoor run*.

‡ *Distribution of herd size among all 2,600 farms Q1 = 1–8, Q2 = 9–14, Q3 = 15–23, Q4 = 24–343 cows*.

### Concourse and Q Set Design

To provide participants with the means to deliver a satisfactory model of their viewpoint, the first stage of a Q methodological study includes “an attempt to survey, as far as possible, the field of what is sayable about the issue of concern” (44). This collection of information is termed concourse (56). Motives potentially impacting on farmers' decisions in the context of improving dairy cow health and welfare were predominantly extracted from scientific publications in the field of animal welfare and farmer attitude. Additionally, online video platforms were searched for interviews with farmers which served in building the concourse. The process of collecting statements was continued until only few new ideas emerged (47). The resulting collection of 344 opinion statements was condensed to a representative sample of manageable size, the Q set. To this end, recurring themes of motives were identified in an open and inductive coding process utilized in qualitative content analysis (57). Terms to create themes were either taken verbatim (e.g., pain) or paraphrased according to the researchers' understanding (e.g., job satisfaction). Iterating this process generated 40 categories (e.g., farmer work satisfaction, profitability, expectations of society, moral obligations toward the animal etc.), each containing several replicates of related statements. Inclusion of statements in the Q set was based on the considerations that (i) each category was represented in the final selection and (ii) selected statements were easy to understand and, as far as possible, attributable to only one category. To ensure clarity and to test the general suitability of the selected statements, the sorting procedure was piloted on 3 university employees and one dairy farmer. The pilot phase led to a simplification of some statements and the replacement of others in order to increase understandability. Furthermore, the category “connection between animal welfare and organic agriculture” was removed because not all participants produced according to organic standards, yielding a final Q set of appropriate size (46, 47) containing 39 statements.

### Sorting Procedure and Post-sort Interview

**Figure 1:**
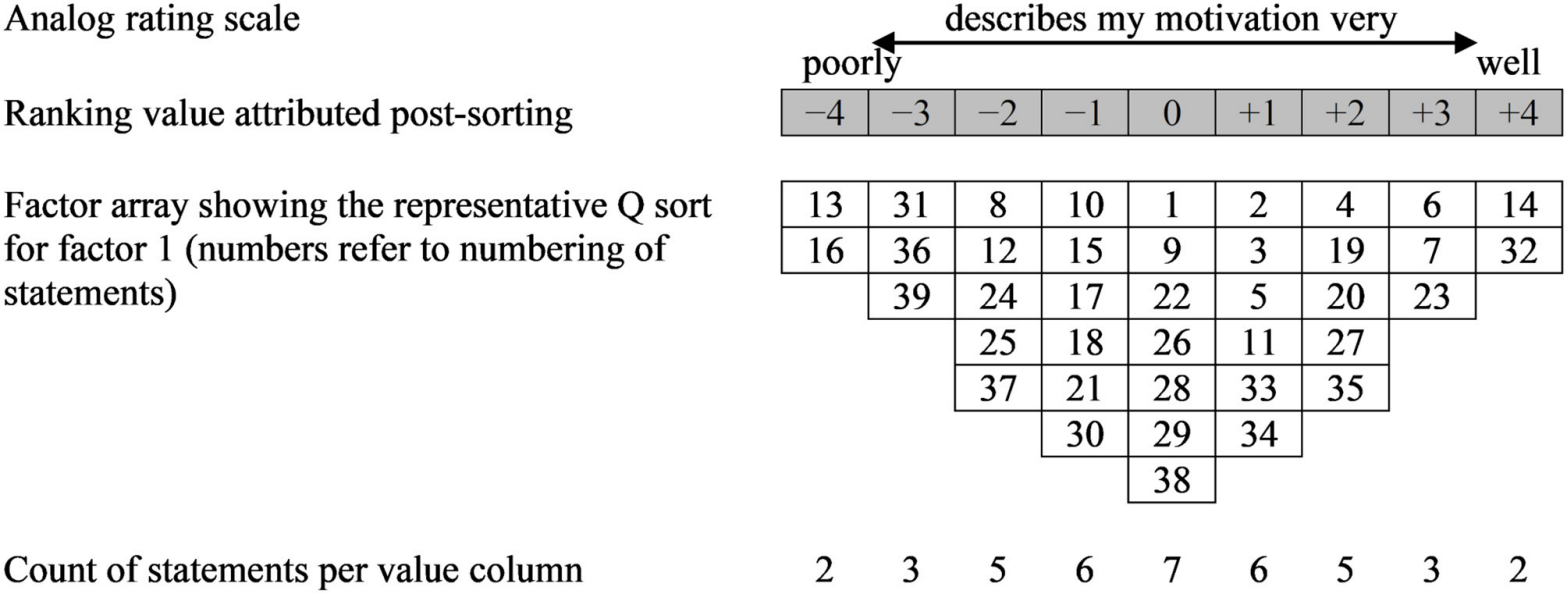
Design of forced choice quasi-normal distribution grid exemplifying the representative Q sort for factor 1.

The data collection process during the farm visits, which lasted for 1.5 h on average, essentially followed recommendations of Watts and Stenner (46). After a short oral introduction to the study goal and the general sequence of data collection, participants were instructed to assign each of the 39 statements a position along an analog rating scale answering the question “What motivates you to ensure that your cows are well?” (see Figure 1). The predefined quasi-normal distribution grid ranged from “describes my motivation very poorly” to “describes my motivation very well.” By positioning all statements relative to each other, farmers were allowed to provide a comprehensive model of their subjective viewpoint on the above question. To facilitate the sorting process, participants were firstly directed to group the Q set into three piles; one for statements that they felt described their motivation well, one for statements they perceived as describing their motivation poorly and a last one for statements they felt indifferent about. Farmers were encouraged to inquire about statements that were unclear. If this was the case, LM explained the researchers' understanding of it, however, trying not to impose a specific meaning on it. The participants were then asked to sort the statements into the grid, starting with any of the three piles. After putting the statements into place, participants were asked to revise the whole sort and correct allocations where deemed necessary. Each column was then assigned a numerical ranking value between −4 to +4 including 0. This step is required for the analysis, but was carried out post-sorting to avoid an impact of the numerical values on the distribution of the statements. Subsequently, in a post-sort interview comments on the statements that participants felt strongly about (sorted between −4 to −2 and between +4 to +2, respectively) were audio-recorded, explaining what the particular statement meant to them and why they had assigned it that position on the rating scale. Also, participants were encouraged to comment on any other issues they wanted to mention (e.g., missing aspects, perception of the method). However, the methodology itself was not discussed in detail during the farm visits.

### Statistical Analysis and Factor Interpretation

One of the 35 participants was excluded from further analysis as the result of the sorting procedure was mistakenly not recorded. Each of the remaining 34 Q sorts was correlated with each other (Software PQMethod, Release 2.35) calculating the Pearson product-momentum correlation coefficient $r=1-\frac{\sum d^{2}}{k}$ where *d* equals the difference between the ranking values of a given statement for the pair of participants to be compared and the constant *k* = 2*Ns*^2^ with *N* = 39 statements and the variance *s*^2^, which, due to the forced distribution, is equal for all Q sorts. The resulting 34 × 34 correlation matrix, with theoretical values ranging between −1 to +1, provides the raw data for the subsequent centroid factor extraction. A correlation coefficient of *r* = +1 would indicate two identical sorts, whereas *r* = −1 would describe two Q sorts being exactly the opposite of one another and *r* = 0 would indicate complete absence of correlation (47). In accordance with recommendations for Q methodological factor analysis, seven factors were extracted and four retained for the subsequent Varimax rotation. This corresponds with suggested solutions based on different criteria (29, 30) (e.g., Humphrey's rule: 1 factor, Scree Test: 2 factors, Kaiser-Guttman criterion: 5 factors, Brown's magic number seven: 7 factors). A Q sort was accepted as significantly loading at *p* < 0.01 on an extracted factor (see Table 2) if the respective rotated factor loading exceeded this study's significance level of $\pm 2.58*\frac{1}{\sqrt{N}}=0.41$. Q sorts significantly loading on a factor are understood as defining the respective factor and were subsequently used to create factor arrays. A factor array in turn is an ideal-typical Q sort for a factor, achieved by a process of weighted averaging of all significantly loading Q sorts and thus has the appearance of a single Q sort (46, 47). Figure 1 exemplarily shows the factor array of factor 1, for which 8 significantly loading Q sorts were considered. Factor arrays for the remaining three factors can be derived from the factor scores listed in Table 3. Each factor is described in the following section, starting with a short summary of demographic details of significantly loading participants. Statements placed at the outermost ends of the forced choice distribution (+/−4 and +/−3), distinguishing statements (Z-scores of statements differing significantly at *p* < 0.01, see Brown (47) for details) as well as the crib-sheet as suggested by Watts and Stenner (46) were used for factor interpretation. A crib-sheet is established for each factor and contains statements ranked at +/−4 and those ranked higher or lower in the respective factor than in any other. Tied statements were also included. This helps to get an idea of what is (non-)essential for and what distinguishes the respective factor from others. Statement number and respective factor score are given in parenthesis (e.g., 32:+4) to support factor interpretation. In order to illustrate the different viewpoints, short quotations derived from the post-sort interviews are presented.

**Table 2:** Rotated factor loadings, count of defining sorts (factor loading exceeding ± 0.41), explained study variance and correlation between factor scores.

**Farmer-ID (Q sort)**		**F1**	**F2**	**F3**	**F4**
1		0.27	0.08	0.36	0.49^*^
2		0.15	0.40	0.30	0.28
3		0.09	**0.41**	0.24	−0.15
4		0.33	0.05	0.41	0.52
5		0.48	0.15	0.42	0.49
6		0.09	**0.74**	0.13	0.20
7		**0.69**	0.13	0.19	0.32
8		0.13	−0.31	0.06	0.16
9		0.00	0.12	−0.02	**0.67**
10		**0.47**	−0.09	0.37	0.02
11		0.24	0.20	0.22	0.06
12		0.34	0.13	0.55	0.41
13		**0.54**	0.21	0.33	0.13
14		0.12	0.19	**0.49**	0.07
15		0.58	0.13	0.19	0.44
16		**0.66**	−0.02	0.15	0.36
17		**0.63**	0.21	0.29	0.30
18		0.26	0.30	0.20	0.32
19		0.01	**0.76**	0.09	0.22
20		−0.15	0.21	0.27	0.12
21		0.39	0.21	0.29	0.00
22		0.30	0.21	0.39	0.30
23		**0.77**	−0.02	0.29	0.15
24		0.02	−0.02	**0.70**	0.30
25		0.28	0.02	0.18	**0.73**
26		**0.63**	0.07	−0.08	0.04
27		0.39	0.03	**0.48**	0.28
28		0.49	0.17	0.36	0.46
29		0.24	**0.53**	0.18	0.18
30		0.19	0.21	**0.68**	−0.08
31		0.06	0.30	**0.68**	0.07
32		0.41	0.21	−0.08	0.44
33		**0.75**	0.04	−0.07	0.10
34		0.31	**0.62**	0.27	0.17
Count of defining sorts		8	5	5	3
Explained variance in %		16	9	12	10
**Correlation between factor scores**					
F1			0.32	0.38	0.44
F2				0.43	0.30
F3					0.33

* *Defining sorts employed for the calculation of the factor arrays are marked in boldface*.

**Table 3:** Collection of statements (Q set) with respective factor scores.

**Statement**		**Factor scores**			
		**F1**	**F2**	**F3**	**F4**
1	To myself, I can only justify having dairy cows if I provide them with good living conditions.	0^*^	*0*	*0*	*0*
2	When my animals are well, I have to call in the veterinarian less often.	1	0	1	2
3	I have more financial returns when my animals are well.	1	3	−1	−2
4	When my cows are well, I am satisfied after a day's work.	2	1	1	2
5	It makes me proud, when my cows are well.	*1*	*2*	*2*	*2*
6	The wellbeing of my cows and my own wellbeing go hand in hand.	**3**^†^	−2	**4**	0
7	When my cows are well, I have more time for other things.	**3**	−2	0	−1
8	It is not enough to only comply with legal standards.	−2	−2	−4	**1**
9	When my cows are well, I can earn a living with my farm.	**0**	−2	−2	−2
10	I want to make sure that on my farm, all legal requirements regarding animal protection are met.	*−1*	*−1*	*−1*	*−1*
11	My cows are my employees.	1	**0**	**−2**	3
12	Regarding animal welfare, I want to hand over my farm to my successor in a better condition than I have inherited it.	**−2**	1	0	−1
13	My son/my daughter will only take over the farm, if we make improvements regarding animal welfare.	−4	−3	−2	−3
14	My cows faring well makes my daily work easier.	4	0	1	3
15	My cows depend on me.	−1	−3	−3	0
16	I want to show other farmers, that a different form of dairy farming is possible.	−4	−4	**0**	−3
17	Animal welfare is important for sustainable agriculture.	−1	2	3	−1
18	I'm always looking for new ideas to further improve my farm. This is also true when it comes to animal welfare.	−1	**4**	**2**	−1
19	My cows have a right to enjoy a good life.	2	1	2	0
20	When my cows are well, I can produce high quality milk.	2	2	2	0
21	When working with cows, economic concerns must not be the only focus.	−1	0	1	**4**
22	I want the dairy company to know that my cows are well.	0	0	−3	−4
23	When the animals are well, they produce as efficiently as possible.	3	3	**−3**	**0**
24	I am worried about future sanctions imposed by the dairy company.	−2	−1	−4	−4
25	I really don't like it, when someone sees a cow in my barn, that is not doing well.	−2	1	−2	1
26	I am only a good dairy farmer, if my cows are well.	0	1	−1	1
27	Feeling that my cows like and trust me just feels good.	2	−1	0	2
28	A positive image of agriculture in the general public is important for our future as dairy farmers.	0	3	3	1
29	For me, cows are sentient beings, above all.	0	−1	2	3
30	Cows must be able to perform their natural behaviors.	**−1**	2	3	2
31	The societal trend toward animal welfare represents an opportunity for my farm.	−3	−1	0	−2
32	When my animals are well, I feel like I'm doing the right thing.	4	0	1	1
33	When a cow is well, she will grow old and we can rely on her for a long time.	1	1	1	**4**
34	I want to avoid unnecessary suffering, pain and stress for the animals.	**1**	4	4	**−1**
35	When my cows are well, it secures a good milk price or good marketing opportunities for me.	**2**	−3	−1	−2
36	Compared to other farms, I want my cows to be better off.	−3	−1	0	−2
37	Animal welfare as a public concern is gaining more and more importance.	−2	**2**	−2	0
38	I see the cows as part of my family.	0	−2	−1	1
39	I want other dairy farmers to see that my cows are well.	−3	−4	−1	−3

* *Consensus statements (those which do not distinguish between any two factors at p > 0.05) are given in italics (statements: 1, 5 and 10)*.

† *Statements which are distinguishing for the respective factor at p < 0.01 are marked in bold. See Brown (47) for details*.

## Results

Descriptive statistics of the P set as well as allocation of participants to extracted factors F1–F4 clustered by sampling criteria are listed in Table 1. Of the 34 Q sorts, 21 were clearly associated with one of the four extracted factors, which together explained 47% of total study variance with the explained variance ranging between 16 (factor 1) and 9% (factor 2). Seven Q sorts were excluded as they did not load high enough on any factor (non-significant Q sorts 2, 8, 11, 18, 20, 21, and 22) and six Q sorts were excluded as they loaded significantly on more than one factor (confounded Q sorts 4, 5, 12, 15, 28, and 32). Table 2 shows the rotated factor loadings of each participant on the four retained factors, as well as the correlations between the four factor arrays. The highest correlations hold between the factor arrays of factors 1 and 4 and factors 2 and 3, with *r*_S_ = 0.44 (*p* = 0.005) and *r*_S_ = 0.43 (*p* = 0.006), respectively. While the correlations indicate that these combinations of factors share some aspects (and might therefore also be interpreted as alternate manifestations of only two viewpoints), we decided to describe them individually as they each hold interesting particularities.

### Viewpoint 1 Focuses on Instrumental Values of Animal Welfare Such as Economic Revenue but at the Same Time on Job Satisfaction and Work-Life-Balance

Factor 1 explains 16% of total variance. Eight participants (three females aged 42–56 y, five males 31–55 y) loaded significantly on this factor. Four of them produced according to EU organic regulations, three produced haymilk. Five farmers kept their cows tethered. Three of those farms routinely provided access to pasture and two were in transition to either tethering with access to an outdoor run and/or pasture or loose housing. The remaining three farmers kept their cows in loose housing systems, with two of them providing access to pasture. Herd size ranged between 7 and 35 dairy cows, with the dairy operation accounting for 50, 75, and 100% of total household income for four, three, and one farm, respectively.

For participants loading on factor 1, the animals' and the farmer's well-being go hand in hand (6:+3), because “if the cows are well, the farmer is well too” (participant 23 = P23). Taking good care of one's livestock is self-explanatory as the daily work with animals gets easier (14:+4) and the farmer has more time left for other things (7:+3). Referring to non-ambulatory cows, P33 commented: “When I have a sick animal, I have to put on my work clothes and I have to move the animal. *Every three or four hours. This is time consuming*.”

A good welfare state of the cows is perceived as a positive feedback on the stockperson's work with the animals. This feeling of doing the right thing is central to the viewpoint of factor 1 (32:+4). P26 explained: “Then you think: ‘I can keep on running the system in this way, and it'll be fine. If not, they would be sick more often’.” Closely linked to this perception of doing the right thing, is a desire for peace and quiet after work (4:+2), as P23 illustrated: “Then I leave the stable in the evening and I see: ‘I didn't need the vet.’ […] That's a good end to a good day.” On the other hand, when “I have to worry or get upset in the barn, I'll be taking it home with me, and I can't relax.”

As much as for work satisfaction and time management, participants loading on factor 1 also see a clear win-win situation regarding productivity or profitability and AHW. Animals produce as efficiently as possible, when they are well (23:+3). “The healthier a cow is, or the fitter, or the more comfortable she feels in the stable, the more milk she can give. Or the less likely she is to get sick” (P26). When a cow is well, she will produce high quality milk (20:+2) enabling the farmer to earn a good farmgate milk price (35:+2) and make a living off farming (9:+0, cf. other factors). Thus, cows experiencing good welfare will ensure profitable farming (3:+1). Consequently, it is legitimate and needs no justification that economic considerations are in the foreground when handling cows (21:−1).

The importance of a good and trusting relationship between human and animal is emphasized (27:+2) and cows are attributed the right to live a good life (19:+2). Yet, the animal as sentient being capable of suffering is not essential to the viewpoint of factor 1 (29:0, 34:+1). This notion is further supported by a rejection of the concept of natural behavior as an important motivation (30:−1). Comparing loose housed and tethered cows, P26 doubts that “a cow can only be well, when she can move around.”

The public discussion about animal welfare is perceived as a fashion and media hype with little relevance for farmers (28:0, 31:−3, 37:−2). “Animal welfare has always been there” (P23). This comment indicates a mindset, in which livestock farmers have always looked after their animals and in which the issue of animal welfare does not need to be addressed by lay people. The opinion of outsiders carries little weight, because people “who have nothing to do with livestock, don't have a clue” (P33). People experienced in dairy farming recognize a sick cow as something normal, unavoidable, and know that the farmer will already have taken appropriate measures. Participants loading on factor 1 are unwilling to justify their work to outsiders, who do not grasp a dairy farmer's reality for lack of knowledge and insight (25:−2). “They are welcome to express their demands. But I don't care. This is my own responsibility” (P23).

People loading on factor 1 do not seem to perceive a need for change regarding living conditions of dairy cattle, neither on their own farm (12:−2, 13:−4, 18:−1), nor in dairy farming in general (16:−4, 36:−3). P23 sums it up as: “Anyway, there is no other way of dairy farming.”

### Viewpoint 2 Tries to Accommodate Societal Expectations Toward Animal Welfare With Economic Success

Factor 2 explains 9% of total variance. Five participants (one female 52 y, four males 36–54 y) loaded significantly on this factor. Three of them produced according to EU organic regulations and three produced haymilk. The animals were loose housed on three farms, two of which provided access to pasture. Both farms with tie-stalls offered access to pasture. Herd size ranged between 15 and 53 dairy cows and the dairy operation contributed 50, 75, and 100% to the total household income of three, one and one farm, respectively.

Participants loading on factor 2 see a close link between the well-being of animals and their productivity (3:+3, 23:+3), because “with an aching foot, a cow will give less milk” (P6). High performance, in turn, does not necessarily equal good welfare, as P19 stated: “I can produce a good amount of milk, even if my animals are not that well.” Thus, animals are perceived to pursue their own goals, which are not always easy to reconcile with the farmer's intention. Pain and stress, for instance, “cannot always be avoided” (P19; 29:−1) which goes along with putting only minor importance to a feeling of doing the right thing when handling animals (32:0). People in factor 2 emphasize a clear distinction between cows and humans (11:0, 38:−2), and cows are not understood as being dependent on the farmer (15:−3). The human-animal relationship is not pivotal to their expressed viewpoint (27:−1).

Job satisfaction and farmer's well-being are not directly linked to animal welfare (4:+1, 6:−2), as P19 put it: “I do pay attention to the well-being of my animals, but for my own well-being…it takes more than just that [well-faring cows].” Easing labor and saving time are not central as motivations (7:−2, 14:0). Quite contrary, it is perceived as “a lot of work, to make sure the cows are well” as P29 explained.

Participants associated with factor 2 refrain from imposing their own viewpoint on, and comparing themselves with others (16:−4, 39:−4). However, there is an indication of very cautious criticism (36:−1, cf. factor 1), which was more openly expressed in the post-sort interviews. Compared to systems of “mass production” (P19, P29), in which “milk is produced at the very expense of the animal” (P19), they want to provide better conditions on their farms. Such an exploitative management style is considered detrimental to the public's image of agriculture, an issue very important within factor 2 (28:+3). Having outsiders see a sick cow in one's own stable, “is unpleasant, *every time*” (P6; 25:+1). Not only is there a marked sensitivity to public opinion, participants loading on factor 2 may even welcome ideas and demands from society (17:+2, 37:+2). P19 explained that, “[…] once in a while, society draws your attention to something, [and] sometimes you end up thinking: ‘*They are not utterly wrong*’.” The public debate about animal welfare therefore, is cautiously perceived as an opportunity rather than a threat (31:−1, cf. other factors), because “the public is also our customer” and “we have to stand out from the crowd” (P29). At the same time, participants in factor 2 are aware of producing for a market in which “price is paramount” (P34; 35:−3).

In contrast to more intensive husbandry systems, the own form of dairy farming is perceived to avoid stress, pain and suffering for the animals (34:+4) and to permit the performance of natural behavior (30:+2). These properties of a husbandry system ensuring the well-being of cows, are at the same time understood to lead to economic success (P19): “That's a clear case: ‘Profit depends on animal welfare’.” Economic performance and animal health and welfare are perceived to be interdependent in this way, making it unnecessary to put economic concerns in the foreground when handling cows (21:0). Participants loading on factor 2 do see conflicts of goals between farmer and cow, but are confident about their own ability to resolve them. They see a need for change in dairy farming toward better animal welfare, and strive to keep up with new developments (12:+1, 18:+4).

### Viewpoint 3 Emphasizes a High Ethical Standard and Does Not Fear Comparison With Fellow Farmers

Factor 3 explains 12% of total variance. Five participants (all male, 32–65 y) were significantly associated with this factor. Four of them produced according to EU organic regulations keeping cows loose housed. One conventional operation kept cows in tie-stalls. Two farms produced hay milk. All five farms provided access to pasture. Herd size varied between 12 and 38 dairy cows and the dairy operation contributed 25, 50, 75, and 100% to the total household income in one, two, one and one farm, respectively.

Participants loading on factor 3 recognize a cow as an animal “born to move” (P31). Thus, allowing cows to perform their natural behavior is of particular importance in their expressed viewpoint (30:+3). A cow is “not only a means of production” (P31; 29:+2) but a sentient being, entitled to enjoy a good life (19:+2), as P24 illustrated: “*She should have a great life*.” Although cows are perceived as distinct from humans (11:−2, 38:−1) and not dependent on farmers (15:−3), it is vital to avoid unnecessary stress, pain and suffering (34:+4).

Farmers associated with factor 3 disapprove of production systems, in which “the cow only lives 3 to 4 lactations and then she is replaced” (P30) and where “the animal itself doesn't count anymore” (P31). Their own way of dairying is perceived to be different from mainstream farming (16:0, cf. other factors), providing improved living conditions (36:0, cf. other factors) and improved animal welfare through innovation (18:+2). There also is relatively little reluctance to compare themselves to other farmers and to present their own approach to dairy farming as different or even superior (39:−1).

A desire to strike a balance between economic and other, ethical considerations is expressed (21:+1), as P24 explains: “[…] profit, sure, yes. One has to make a living. But it's not everything. *Not at any price*.” Consequently, productivity and profitability are rejected as motivations to invest effort into improving animal welfare (3:−1, 23:−3). P30 illustrated: “As an organic farmer, you cannot just go for the money. You have to kind of live this and say: ‘Well, even though this cow might not be very profitable any more, she can stay a while longer, and *that's it*’.”

Saving time and easing labor is not vital to the viewpoint of factor 3 (7:0, 14:+1), because “if you are a dairy farmer, the animals come first” (P24). Personal well-being is nevertheless important and is considered to go hand in hand with animal welfare (6:+4). As P31 explained, “when you notice a sick calf in the evening, you won't get a good night's sleep.”

Though demands of society regarding animal welfare rather pose an opportunity than a threat (31:0, cf. other factors), interference of outsiders or business partners is clearly rejected as a motivation to ensure animal welfare (8:−4, 22:−3, 24:−4, 25:−2, 37:−2). Commenting on statement 37, P24 clarified: “as a farmer, it's first of all your own responsibility to take good care of the animals. You mustn't let others push you around too much, or they will start to command what you have to do.” Animal welfare contributing to sustainable agriculture (17:+3) is as important as a positive image of agriculture in society, since “we always have to justify ourselves for keeping livestock” (P30; 28:+3).

### Viewpoint 4 Strives to Share a Good Life With Cows

Factor 4 explains 10% of total variance. Three participants (one female 57 y, two males 32 and 44 y) were significantly associated with this factor. All of them produced according to EU organic regulations with loose housing and access to pasture. Additionally, two of them produced haymilk. Operation size was 6, 7, and 22 dairy cows, respectively. The dairy operation contributed 25 and 50% to total household income in 2 and 1 farm, respectively.

For participants loading on factor 4, cows have to be treated with “respect and appreciation” (P25). Animals are perceived as sentient beings, or even “persons” (P9; 11:+3, 29:+3, 38:+1). The animals' dependence on humans makes it necessary to care for them (15:0, cf.: other factors) and to give them the opportunity to perform natural behavior (30:+2). A trusting relationship with the animals is central to the viewpoint of factor 4 and leads to both emotional and work-related benefits. P9 explains: “Stroking calves is not only nice and calming. The animals are much easier to handle once they know that I mean them no harm. […] If a cow sees me as her enemy, […] we both have an unpleasant life” (4:+2, 27:+2). Easing daily labor (14:+3) is an important motivation in factor 4, with an impact on personal, emotional well-being, as P25 described: “When a cow has problems with her claws, you will be confronted with this every day […]. That's not a good feeling […] and you will keep asking yourself: ‘Why?’.” Legal requirements on animal protection are considered an absolute must but neither sufficient nor satisfactory (8:+1). Surprisingly, avoiding unnecessary pain, stress and suffering is rejected as a motivation (34:−1). This is a seemingly contradictory result, which P9 explains as follows: “When I read this, I'm thinking: ‘*Too much has gone wrong already*’.”

For participants loading on factor 4, a positive correlation between farm profitability and animal welfare exists “only to some extent and is maybe just not that important. […] If, for example, a calf stays longer on my farm, I don't make more profit, but the animals are better off” (P25). Profitability and productivity are rejected as motivations (3:−2, 23:0). P9 explained: “it's not all about the figures. Of course, if you would reach your limits [of economic viability], then you would have to look at the figures. But it's not what you think of during your daily work in the stable. You don't think: ‘I will brush this cow now, maybe that'll make me *another 3 cents*.’.”

Reducing expenses for veterinary treatment and extending the productive life-span of cows (2:+2, 33:+4) fits better into the viewpoint of factor 4 farmers, because “if the cows grow old, the economic part usually turns out to be just fine” (P25). However, economic considerations seem to be less important than emotional concerns. P9 explains: “There is this saying that old cows and young hens make a farmer rich […]. But the thing is, the older a cow gets, the more you get used to her. You just like her because of the many years you have spent together.” Apparently, participants loading on factor 4 feel that in some situations “it is necessary to put aside economic thoughts. Sometimes it's better to focus on personal, and on emotional matters” (P25; 21:+4).

While meeting their own high ethical standards is important to participants loading on factor 4, they are reluctant to act as role models or to compare themselves to other farmers (16:−3, 36:−2, 39:−3). Demands and opinions of society and other stakeholders regarding animal welfare seem to be rather irrelevant to participants in this group (25:+1, 28:+1, 31:−2, 35:−2, 37:0), and the dairy company is clearly rejected as a relevant reference point concerning animal welfare (22:−4, 24:−4). P9 boiled it down to: “I'd rather do it for the cow, than for the recognition.”

## Discussion

Using Q methodology, we investigated how Austrian dairy farmers assessed a set of potentially motivating statements regarding the improvement of AHW. We identified and discriminated between four viewpoints, showing that (i) there are important differences between individual farmers in terms of what they perceive as motivating to improve AHW, and, despite these individual differences, that (ii) farmers can be subsumed in distinct viewpoints according to their motivational pattern regarding the improvement of AHW. To our knowledge this is the first study employing Q methodology to investigate farmer motivation to improve dairy cow welfare. Directly comparable literature is therefore lacking. Still, related research is available in order to allow a comprehensive discussion of our findings.

### The Complexity of Farmer Motivation Merits Detailed Distinction

The understanding of what defines good AHW and the emphasis put on intrinsic and instrumental values of AHW allows a rough classification of the identified points of view. Participants sharing Viewpoint 1 are predominantly motivated by instrumental values and seem to understand animal welfare mostly in terms of biological functioning. The animals' affective state and natural behavior were not important for this group. In contrast, Viewpoints 3 and 4, respectively, are characterized by an understanding of AHW that also incorporates concepts of affective state and natural behavior. Participants loading on these two factors describe themselves as motivated mainly by intrinsic values of AHW related to empathy and moral obligations toward the sentient animal. Viewpoint 2 is particularly interesting, as it falls between these two large groups. On the one hand, it is characterized by an understanding of animal welfare that goes beyond biological functioning. On the other hand, participants sharing Viewpoint 2 emphasize a legitimate connection between business goals and AHW. These findings are largely in line with Bock et al. (58), who described two groups of European pig farmers differing in their perception and definition of animal welfare. One group thought of animal welfare mostly in terms of basic biological needs, seeing production performance (e.g., growth) and animal health as the best indicators of good animal welfare. Animal welfare was important to them, because of its impact on zoo-technical performance and economic success. A second group, mostly consisting of farmers participating in specific animal welfare or organic farm assurance schemes, understood animal welfare in terms of the animal's ability to express natural behavior and emphasized freedom and comfort of the animals. They valued animal welfare both for its impact on production and business, and for ethical reasons connected to the animal as a sentient being. A very similar classification of farmers' understanding and valuation of AHW was obtained by Kauppinen et al. (33) (intrinsic and instrumental value) and Austin et al. (59) (business and welfare orientation), together reflecting the four viewpoints found in the present study.

### Instrumental and Intrinsic Values of AHW

The results of our study show that not all dairy farmers are motivated by the same drivers to improve AHW. Instrumental values are particularly strong motivational drivers for Viewpoint 1 but also present in Viewpoint 2. For example, the effect of good AHW on efficiency of milk production and on milk quality is important. However, Viewpoint 2 focuses on economic performance, thus corresponding with the finding of Hansson and Lagerkvist (31) that the most important instrumental values of AHW for Swedish dairy farmers were related to the economic performance of the farm. Viewpoint 1 puts much more emphasis on job satisfaction, a good work environment and reducing time for herd-related work, thus freeing up time for other tasks or leisure. However, when it comes to the other instrumental values of good AHW as outlined above, there are important differences between our results and those of Hansson and Lagerkvist (31). In the latter study, freeing up time and experiencing a good work environment was not important to farmers in the overall ranking. This is in contrast to the strong emphasis put on these aspects by Viewpoint 1 in our study and might be attributed to the fact that Hansson and Lagerkvist derived one overall ranking of values for all participating farmers. Differences between individuals may thus have been masked. In this context, it is interesting to note that, performing a (by-item) principal component analysis the value statements “To make sure that my dairy cows are healthy, so that I have time available to do other things” and “To make sure that my dairy cows are kept in such a way that I can earn my living from my business” loaded on the same component (31). While it is important to remember that this is not the same as a factor in Q methodology (which is based on correlations between individuals), it is still noteworthy that these two instrumental values also go together in our study: they were both quite important for Viewpoint 1.

Also the results of Valeeva et al. (20) closely resemble Viewpoint 1 in our study. Investigating the motivation of dairy farmers to improve mastitis management, they compared a hypothetical milk price premium to a penalty scenario. While avoiding animal pain and suffering (an intrinsic value of AHW) did play a role in the decision to address udder health problems, the most important motivational drivers revolved around instrumental values. Based on the perceived importance of eight selected motivational drivers, farmer behavior was most strongly affected by the expected effect of improved udder health on job satisfaction (“intangible feelings of farmer pleasure” e.g., doing the right thing), the overall situation on the farm (practical benefits such as “making daily work easier”) and economic performance.

Viewpoints 3 and 4 described themselves as motivated mainly by intrinsic values of AHW. Also Hansson and Lagerkvist (31) found that intrinsic values (e.g., a feeling of happiness associated with treating cows well, absolute rights attributed to animals) were stronger motivational drivers of farmer behavior than instrumental values (e.g., profitability, being able to continue the business). However, this general finding was not confirmed in our study, since participants sharing Viewpoint 1, for example, described themselves as predominantly motivated by instrumental values of AHW.

Valeeva et al. (20) also investigated possible individual differences between farmers in relative importance attributed to the eight motivational drivers. Applying cluster analysis to identify groups of farmers sharing similar motivational patterns, farmers differed in what they perceived as most motivating. The groups differed, among other aspects, in the relative importance they attributed to the different instrumental values offered in the study (job satisfaction, work environment and economic performance), i.e., in the nature of instrumental benefits they valued most (cf. Viewpoints 1 and 2). The authors concluded, that when trying to stimulate behavior change in the context of mastitis management, it is important (i) to take into account individual differences between farmers in motivational pattern (i.e., not everyone is motivated by the same things) and (ii) to pay special attention to which type of instrumental benefits is most important to the individual in question, i.e., to go beyond monetary benefits and take into account “soft” or practical issues such as job satisfaction and work environment. These findings are very much in line with our results which show that individual differences go beyond a simple dichotomy (intrinsic vs. instrumental), and that in order to thoroughly understand farmer motivation in the context of AHW, it is worthwhile to distinguish between different farmer viewpoints.

### Social Norms and Farmers' Relationship to Outside Reference Groups

The perception of what relevant other people do or expect one to do, is generally recognized as strong driver of human behavior (60). Howley (40) argues, that farmers seek to balance economic, social and lifestyle goals and cannot be understood as mere profit maximizers. For understanding and changing farmer behavior, it is therefore of particular interest how farmers relate to outside reference groups (e.g., veterinarians, herd health advisors) as social norm. While Viewpoint 2 is characterized by an outward orientation toward society and market and an attentiveness to what outsiders think, Viewpoints 1 and 3 reject interference from outside parties, especially from groups perceived to have limited knowledge about the practical reality of dairy farming. In accordance with the outward oriented Viewpoint 2, Hansson and Lagerkvist (31) found that the recognition by reference groups, both inside and outside the dairy sector, was rated highly among the benefits of good animal welfare. Similar to Viewpoint 2 in the present study, this openness toward society was related to entrepreneurial or marketing considerations, aimed at being able to meet changing consumer demands. In another study (25), the same authors found recognition by consumers and authorities to be valued by farmers, especially for marketing reasons. Participating farmers saw a positive impression on outside reference groups as a necessary precondition for sustained sales in the long run. These results, and the viewpoint of factor 2 in the present study, suggest that being able to fulfill expectations of outside reference groups in society may be important at least to some farmers. However, Valeeva et al. (20) showed that dairy farmers in the specific context of mastitis management attributed little importance to recognition for a job well done (e.g., awards or articles published in specialist magazines) or improving the public image of dairy production. Focusing on the reference group of fellow farmers, little importance is attributed by Viewpoints 1, 2 and 4, respectively and any comparison to other farmers is rejected. Only Viewpoint 3 shows comparably little reluctance to benchmarking with other farmers. Applying a Theory of Planned Behavior framework, Jones et al. (15) found that farmers' beliefs about actions and expectations of important social reference groups, such as peer farmers, have very limited influence on intentions of dairy farmers to adopt additional herd health measures. This is in accordance with Viewpoints 1, 2, and 4. They conclude that installing successful others as role models or benchmarks is unlikely to help in achieving behavior change. Fellow farmers as social reference group were also found to have only little impact on farmers' decision to join a hypothetical dairy health program regarding Bovine Virus Diarrhea (38). With regard to mastitis management, Jansen et al. (61) found more pronounced differences when investigating the so-called “hard to reach”-farmer. Farmers described as such did not constitute a homogeneous group but could be divided into 4 distinct categories based on data from semi-structured interviews. They differed greatly in their orientation toward their social environment and their trust in information received from outside sources. While some farmers were found to be open toward outside groups (e.g., other farmers) and information sources, others were inward oriented and focused mainly on their own farm. Particularly, the “Reclusive Traditionalists” as termed by Jansen et al. (61), characterized by an inward orientation, a dislike for interference from outside and a reluctance to compare themselves with other farmers, share these properties with Viewpoint 1 in our study. In contrast Viewpoint 2 in our study, which is open explicitly to consumer demands resembles characteristics of the so-called “Proactivists,” which were described as well informed and interested in new development.

### Limitations of the Study

Q Methodology was considered especially promising for the investigation of farmers' viewpoints toward the improvement of animal welfare, as it allows a thorough comparison of comprehensive representations of individuals by balancing different statements along a qualitative rating scale in combination with rigorous statistical correlation and factor extraction techniques. In contrast, utilizing an R methodological approach does not allow to capture holistic images of individuals (46). However, a potential limitation of this study lies in the construction of the concourse, i.e., how we compiled the collection of possible motivating aspects of good animal health and welfare, which was subsequently reduced to form the set of statements to be sorted (Q set). The better this set of statements represents the diversity of ideas present among the studied population, the easier it is for participants to express their viewpoint accurately (46). Building the collection on scientific literature and online sources alone may have led to some ideas not being present in the set of statements, limiting participants in the expression of their viewpoint. Collecting material directly from natural speech of farmers, i.e., in-depth qualitative interviews or focus groups, might have added additional information. Apart from this limitation particular to our approach, it is important to acknowledge, that the researcher's choice of which statements to provide for sorting obviously limits the participants in expressing their viewpoints and influences the emerging factors and their interpretation. Despite possible shortcomings of the offered set of statements, the clear differences described above show that participants in our study were able to use the statements to express distinct viewpoints. Furthermore, non-response bias may have occurred to some degree, as 39 of the initially 73 selected participants refused to take part in our study and it was particularly difficult to recruit conventional farmers. Non-response bias is especially probable if there is an important relationship between a person's decision to participate (or not) and the subject the researchers want to study (62). In our case, a person's attitude toward animal health and welfare may well affect their decision (not) to participate in our study (especially seen against the backdrop of tense societal discussion on this topic). To reduce the bias introduced by self-selection, a priori selection criteria were defined which we expected to be related to differing attitudes toward animal health and welfare. We continued recruitment until the obtained participant group was balanced with regard to these selection criteria. Another possible source of bias in our study is the effect of social desirability during the sorting and interview phase. Participants may perform or report behavior according to perceived expectations of others (63). To reduce the effect of social desirability on the behavior of participants, LM visited all participants at their homes, where we expected them to feel comfortable and tried to establish an atmosphere of trust during sorting and interview.

## Conclusion

As farmers are the ultimate decision-makers, understanding their motivation for improving animal health and welfare and knowing about respective differences is of great importance for facilitating change and planning intervention strategies. Using Q methodology, we were able to draw high resolution images of different farmer typologies. We argue that it is not important what the dominating viewpoint within a population of dairy farmers is, but rather it is essential to identify the diversity of viewpoints and find a quick and easy way to categorize farmers accordingly. Farm and personal characteristics may indicate how animal health and welfare are defined and valued and further research is suggested to develop a method for rapid classification of farmers according to the identified typologies. Considering these individual differences may allow a more adequate approach to induce behavior change and consequently enables advisors to specifically address leverage points with a high chance of farmer compliance. For example, communication strategies and incentives could in future be tailored to the viewpoints identified, thus helping extension specialists or veterinarians in taking on a proactive role in the process of improving AHW. Such knowledge can also be integrated in the training and education of advisors.

## Data Availability Statement

The raw data supporting the conclusions of this article will be made available by the authors, without undue reservation.

## Ethics Statement

Ethical review and approval was not required for the study on human participants in accordance with the local legislation and institutional requirements. The patients/participants provided their written informed consent to participate in this study.

## Author Contributions

JS and CW conceived the study. LM and JS contributed equally to this work and drafted the manuscript. All authors edited the manuscript, contributed to manuscript revision, and approved the submitted version.

## Conflict of Interest

The authors declare that the research was conducted in the absence of any commercial or financial relationships that could be construed as a potential conflict of interest.

## References

[1] Solano L Barkema HW Pajor EA Mason S LeBlanc SJ Zaffino Heyerhoff JC. Prevalence of lameness and associated risk factors in Canadian holstein-friesian cows housed in freestall barns. J Dairy Sci. (2015) 98:6978–91. 10.3168/jds.2015-9652 26254526

[2] Rutherford KMD Langford FM Jack MC Sherwood L Lawrence AB Haskell MJ. Lameness prevalence and risk factors in organic and non-organic dairy herds in the United Kingdom. Vet J. (2009) 180:95–105. 10.1016/j.tvjl.2008.03.015 18462961

[3] Leach KA Whay HR Maggs CM Barker ZE Paul ES Bell AK. Working towards a reduction in cattle lameness: 2. understanding dairy farmers' motivations. Res Vet Sci. (2010) 89:318–23. 10.1016/j.rvsc.2010.02.017 20413137

[4] Alvergnas M Strabel T Rzewuska K Sell-Kubiak E Claw disorders in dairy cattle: effects on production, welfare and farm economics with possible prevention methods. Livestock Sci. (2019) 222:54–64. 10.1016/j.livsci.2019.02.011

[5] Jansen J van den Borne BHP Renes RJ van Schaik G Lam TJGM Leeuwis C. Explaining mastitis incidence in Dutch dairy farming: the influence of farmers' attitudes and behaviour. Prev Vet Med. (2009) 92:210–23. 10.1016/j.prevetmed.2009.08.015 19800700

[6] Jamali H Barkema HW Jacques M Lavallée-Bourget E-M Malouin F Saini V. Invited review: incidence, risk factors, and effects of clinical mastitis recurrence in dairy cows. J Dairy Sci. (2018) 101:4729–46. 10.3168/jds.2017-13730 29525302

[7] Schukken YH Grommers FJ van de Geer D Erb HN Brand A. Risk factors for clinical mastitis in herds with a low bulk milk somatic cell count. 1. data and risk factors for all cases. J Dairy Sci. (1990) 73:3463–71. 10.3168/jds.S0022-0302(90)79045-5 2099368

[8] Nielsen BH Thomsen PT Sørensen JT. Identifying risk factors for poor hind limb cleanliness in Danish loose-housed dairy cows. Animal. (2011) 5:1613–9. 10.1017/S1751731111000905 22440353

[9] Zurbrigg K Kelton D Anderson N Millman S. Tie-stall design and its relationship to lameness, injury, and cleanliness on 317 ontario dairy farms. J Dairy Sci. (2005) 88:3201–10. 10.3168/jds.S0022-0302(05)73003-4 16107410

[10] Kester E Holzhauer M Frankena K. A descriptive review of the prevalence and risk factors of hock lesions in dairy cows. Vet J. (2014) 202:222–8. 10.1016/j.tvjl.2014.07.004 25201250

[11] Ekman L Nyman A-N Landin H Waller KP. Hock lesions in dairy cows in freestall herds: a cross-sectional study of prevalence and risk factors. Acta Vet Scand. (2018). 60:47. 10.1186/s13028-018-0401-9 30103790 PMC6090646

[12] Thomsen PT Kjeldsen AM Sørensen JT Houe H Ersbøll AK. Herd-level risk factors for the mortality of cows in Danish dairy herds. Vet Rec. (2006) 158:622–6. 10.1136/vr.158.18.622 16679480

[13] Reimus K Alvåsen K Emanuelson U Viltrop A Mõtus K. Herd-level risk factors for cow and calf on-farm mortality in Estonian dairy herds. Acta Vet Scand. (2020) 62:15. 10.1186/s13028-020-0513-x 32164740 PMC7068997

[14] Oltenacu PA Algers B. Selection for increased production and the welfare of dairy cows: are new breeding goals needed? Ambio. (2005) 34:311–5. 10.1579/0044-7447-34.4.311 16092261

[15] Jones PJ Sok J Tranter RB Blanco-Penedo I Fall N Fourichon C. Assessing, and understanding, European organic dairy farmers' intentions to improve herd health. Prev Vet Med. (2016) 133:84–96. 10.1016/j.prevetmed.2016.08.005 27720030

[16] Archer S Bell N Huxley J Lameness in UK dairy cows: a review of the current status. Practice. (2010) 32:492–504. 10.1136/inp.c6672

[17] Green MJ Leach KA Breen JE Green LE Bradley AJ. National intervention study of mastitis control in dairy herds in England and wales. Vet Rec. (2007) 160:287–93. 10.1136/vr.160.9.287 17337605

[18] Tremetsberger L Winckler C Effectiveness of animal health and welfare planning in dairy herds: a review. Anim Welfare. (2015) 24:55–67. 10.7120/09627286.24.1.055 26233459

[19] Tremetsberger L Leeb C Winckler C. Animal health and welfare planning improves udder health and cleanliness but not leg health in Austrian dairy herds. J Dairy Sci. (2015) 98:6801–11. 10.3168/jds.2014-9084 26233459

[20] Valeeva NI Lam TJGM Hogeveen H. Motivation of dairy farmers to improve mastitis management. J Dairy Sci. (2007) 90:4466–77. 10.3168/jds.2007-0095 17699068

[21] Bell NJ Bell MJ Knowles TG Whay HR Main DJ Webster AJF. The development, implementation and testing of a lameness control programme based on HACCP principles and designed for heifers on dairy farms. Vet J. (2009) 180:178–88. 10.1016/j.tvjl.2008.05.020 18694651

[22] Higgins H Green M Madouasse A Facilitating change in herd health. In: Green M editor. Dairy herd health. Wallingford, CT: CABI (2012). p. 11–33. 10.1079/9781845939977.0011

[23] Atkinson O Communication in farm animal practice 1. farmer-vet relationships. Practice. (2010) 32:114–7. 10.1136/inp.c836

[24] Kristensen E Jakobsen EB. Challenging the myth of the irrational dairy farmer; understanding decision-making related to herd health. N Z Vet J. (2011) 59:1–7. 10.1080/00480169.2011.547162 21328151

[25] Hansson H Lagerkvist CJ Identifying use and non-use values of animal welfare: evidence from Swedish dairy agriculture. Food Policy. (2015) 50:35–42. 10.1016/j.foodpol.2014.10.012

[26] McInerney J Animal Welfare, Economics and Policy Report on a Study Undertaken for the Farm & Animal Health Economics Division of Defra. Exeter (2004)

[27] Bruijnis MRN Hogeveen H Stassen EN. Measures to improve dairy cow foot health: consequences for farmer income and dairy cow welfare. Animal. (2013) 7:167–75. 10.1017/S1751731112001383 23031420

[28] Bruijnis MRN Hogeveen H Stassen EN. Assessing economic consequences of foot disorders in dairy cattle using a dynamic stochastic simulation model. J Dairy Sci. (2010) 93:2419–32. 10.3168/jds.2009-2721 20494150

[29] Hogeveen H Huijps K Lam TJGM. Economic aspects of mastitis: new developments. N Z Vet J. (2011) 59:16–23. 10.1080/00480169.2011.547165 21328153

[30] Whay HR Main DCJ Improving animal welfare: practical approaches for achieving change. In: Grandin T editor. Improving Animal Welfare: A Practical Approach / Edited by Temple Grandin. Wallingford, CT: CABI (2010) p. 227–51.

[31] Hansson H Lagerkvist CJ. Dairy farmers' use and non-use values in animal welfare: Determining the empirical content and structure with anchored best-worst scaling. J Dairy Sci. (2016) 99:579–92. 10.3168/jds.2015-9755 26547638

[32] Ajzen I. The theory of planned behavior. Org Behav Hum Decision Process. (1991) 50:179–211. 10.1016/0749-5978(91)90020-T21929476

[33] Kauppinen T Vainio A Valros A Rita H Vesala KM Improving animal welfare: qualitative and quantitative methodology in the study of farmers' attitudes. Anim Welfare. (2010) 19:523–36. Available online at: https://www.ingentaconnect.com/contentone/ufaw/aw/2010/00000019/00000004/art00015

[34] Kauppinen T Valros A Vesala KM Attitudes of dairy farmers toward cow welfare in relation to housing, management and productivity. Anthrozoös. (2013) 26:405–20. 10.2752/175303713X13697429463718

[35] de Lauwere C van Asseldonk M van ‘t Riet J de Hoop J Ten PE Understanding farmers' decisions with regard to animal welfare: the case of changing to group housing for pregnant sows. Livestock Sci. (2012). 143:151–61. 10.1016/j.livsci.2011.09.007

[36] Dutton-Regester KJ Wright JD Rabiee AR Barnes TS. Understanding dairy farmer intentions to make improvements to their management practices of foot lesions causing lameness in dairy cows. Prev Vet Med. (2019) 171:104767. 10.1016/j.prevetmed.2019.104767 31518830

[37] Borges JAR Domingues CHdF Caldara FR Rosa NPd Senger I Guidolin DGF. Identifying the factors impacting on farmers' intention to adopt animal friendly practices. Prev Vet Med. (2019) 170:104718. 10.1016/j.prevetmed.2019.104718 31421489

[38] Lauwere C de van Asseldonk M Bergevoet R Bondt N Understanding decision-making of dairy farmers with regard to participation in a dairy health programme. Livestock Sci. (2020) 239:104061 10.1016/j.livsci.2020.104061

[39] Heong KL Escalada MM, editors. Pest Management of Rice Farmers in Asia. Manila (Philippines): IRRI (1997).

[40] Howley P The happy farmer: the effect of nonpecuniary benefits on behavior. Am J Agricult Econ. (2015) 97:1072–86. 10.1093/ajae/aav020

[41] Addams H Q Methodology. In: Addams H Proops JLR editors. Social Discourse and Environmental Policy: An Application of Q Methodology. Cheltenham, Northampton, MA: Edward Elgar Pub (2001) p. 14–40.

[42] Brown SR A primer on q methodology. Operant Subject. (1993) 16:91–138.

[43] Stenner PHD Cooper D Skevington SM. Putting the Q into quality of life; the identification of subjective constructions of health-related quality of life using Q methodology. Soc Sci Med. (2003) 57:2161–72. 10.1016/S0277-9536(03)00070-4 14512246

[44] Stenner PHD Dancey CP Watts S. The understanding of their illness amongst people with irritable bowel syndrome: a Q methodological study. Soc Sci Med. (2000) 51:439–52. 10.1016/S0277-9536(99)00475-X 10855930

[45] Zabala A Pascual U. Bootstrapping Q methodology to improve the understanding of human perspectives. PLoS ONE. (2016) 11:e0148087. 10.1371/journal.pone.0148087 26845694 PMC4742059

[46] Watts S Stenner P Doing Q Methodological Research: Theory, Method and Interpretation. Los Angeles, CA: SAGE (2012). 10.4135/9781446251911

[47] Brown SR Political Subjectivity: Applications of Q Methodology in Political Science. New Haven; London: Yale University Press (1980)

[48] Vecchio Y Pauselli G Adinolfi F. Exploring attitudes toward animal welfare through the lens of subjectivity-an application of q-methodology. Animals. (2020) 10:1364. 10.3390/ani10081364 32781735 PMC7460027

[49] Baker RM. Economic rationality and health and lifestyle choices for people with diabetes. Soc Sci Med. (2006) 63:2341–53. 10.1016/j.socscimed.2006.06.007 16875768

[50] Truong DB Binot A Peyre M Nguyen NH Bertagnoli S Goutard FL. A Q method approach to evaluating farmers' perceptions of foot-and-mouth disease vaccination in Vietnam. Front Vet Sci. (2017) 4:95. 10.3389/fvets.2017.00095 28695123 PMC5483627

[51] Kristensen E Enevoldsen C. A mixed methods inquiry: how dairy farmers perceive the value(s) of their involvement in an intensive dairy herd health management program. Acta Vet Scand. (2008) 50:50. 10.1186/1751-0147-50-50 19091134 PMC2628344

[52] Zanoli R Cuoco E Barabanova Y Mandolesi S Naspetti S Using Q methodology to facilitate the establishment of the 2030 vision for the EU organic sector. Org Agr. (2018) 8:265–73. 10.1007/s13165-018-0207-0

[53] Walder P Kantelhardt J The environmental behaviour of farmers – capturing the diversity of perspectives with a q methodological approach. Ecol Econ. (2018) 143:55–63. 10.1016/j.ecolecon.2017.06.018

[54] Brodt S Klonsky K Tourte L Farmer goals and management styles: implications for advancing biologically based agriculture. Agricult Syst. (2006) 89:90–105. 10.1016/j.agsy.2005.08.005

[55] MacCallum R Widaman K Zhang S Hong S. Sample size in factor analysis. Psychol Methods. (1999) 4:84–99. 10.1037/1082-989X.4.1.8426822184

[56] Stephenson W Concourse theory of communication. Communication. (1978) 3:21–40. 10.1109/TPC.1978.6592443

[57] Kuckartz U Qualitative Inhaltsanalyse. Methoden, Praxis, Computerunterstützung. 4. Auflage. Weinheim, Basel: Beltz Juventa (Grundlagentexte Methoden) (2018).

[58] Bock BB van Huik MM Animal welfare: the attitudes and behaviour of European pig farmers. Br Food J. (2007) 109:931–44. 10.1108/00070700710835732

[59] Austin EJ Deary IJ Edwards-Jones G Arey D Attitudes to farm animal welfare. J Ind Diff. (2005) 26:107–20. 10.1027/1614-0001.26.3.107

[60] Cialdini RB Descriptive social norms as underappreciated sources of social control. Psychometrika. (2007) 72:263–8. 10.1007/s11336-006-1560-6

[61] Jansen J Steuten CDM Renes RJ Aarts N Lam TJGM. Debunking the myth of the hard-to-reach farmer: effective communication on udder health. J Dairy Sci. (2010) 93:1296–306. 10.3168/jds.2009-2794 20172249

[62] Listyowardojo TA Nap RE Johnson A Demographic differences between health care workers who did or did not respond to a safety and organizational culture survey. BMC Res Notes. (2011) 4:328 10.1186/1756-0500-4-328 21899771 PMC3180706

[63] Lusk JL Norwood FB Direct versus indirect questioning: an application to the well-being of farm animals. Soc Indic Res. (2010) 96:551–65. 10.1007/s11205-009-9492-z

