# Supplementary material for: Resource, Collaborator, or Individual Cow? Applying Q Methodology to Investigate Austrian Farmers' Viewpoints on Motivational Aspects of Improving Animal Welfare
Source: Front Vet Sci. 2021 Jan 12;7:607925. doi: 10.3389/fvets.2020.607925 (PMC7873868; doi:10.3389/fvets.2020.607925)
Supplement: Supplementary file 1 [file Table_1.pdf]

Supplementary Table 1: Individual Q sorts of 34 study participants showing ranking values for each of the 39 statements

																			Statement																				
**Q sort**	**1**	**2**	**3**	**4**	**5**	**6**	**7**	**8**	**9**	**10**	**11**	**12**	**13**	**14**	**15**	**16**	**17**	**18**	**19**	**20**	**21**	**22**	**23**	**24**	**25**	**26**	**27**	**28**	**29**	**30**	**31**	**32**	**33**	**34**	**35**	**36**	**37**	**38**	**39**
**1**	0	1	0	1	3	-1	-1	-1	-3	-2	4	2	-3	1	0	-2	-1	-2	2	-1	2	-4	-1	-3	0	0	1	3	2	1	-4	3	1	2	0	0	-2	4	-2
**2**	1	2	1	2	-1	3	-2	1	-1	0	-1	-2	-3	-4	-3	-3	2	4	-2	1	2	-2	0	0	0	0	-1	4	2	3	-1	1	0	3	1	-4	-1	0	-2
**3**	-1	3	3	1	1	-4	1	-3	0	-3	0	0	-4	1	-1	2	-2	4	2	0	-2	0	-1	-3	2	0	-2	4	-2	0	1	-1	3	1	-2	-1	2	-1	2
**4**	-2	-2	-1	0	2	1	-2	1	0	0	2	-3	-4	0	0	-1	1	1	2	2	1	-2	0	-4	-3	-1	3	0	3	2	-1	4	1	3	-2	-3	-1	4	-1
**5**	1	-1	-1	4	1	3	2	-1	0	-1	0	-2	-3	1	-1	-4	1	4	2	0	1	-3	0	-3	-2	0	3	0	2	2	-2	0	2	3	-1	-2	-2	1	-4
**6**	0	-1	1	0	0	-1	-2	-1	-3	-1	0	-1	-2	2	-4	-2	3	3	1	3	1	-1	4	2	2	2	0	4	0	1	-4	0	1	2	-3	-2	1	-3	-2
**7**	2	0	0	1	1	4	1	-2	-1	-1	4	-3	-3	3	-2	-4	1	1	2	2	-2	0	2	-3	-1	1	2	0	0	-1	-2	3	3	-1	0	-4	-1	0	-2
**8**	-1	3	1	1	2	4	3	-1	-2	2	0	-1	-2	1	4	2	1	-2	0	0	3	-3	-2	-1	0	0	-2	-4	-3	2	2	0	1	-3	1	-4	0	-1	-1
**9**	0	3	-2	2	1	-3	-2	2	-2	0	2	-1	-1	0	1	-2	0	-2	0	1	4	-4	2	-4	1	2	3	1	3	0	-1	1	4	-3	-3	-1	0	-1	-1
**10**	0	-1	2	2	2	2	1	-4	0	-2	-3	1	-2	3	3	-1	-3	-2	-1	1	-1	-2	0	-4	-3	0	1	3	4	0	-2	0	2	1	4	1	-1	0	-1
**11**	2	0	-2	1	4	1	-3	0	0	1	-4	-1	-2	-4	2	-1	1	1	3	2	-1	1	0	-1	-2	0	2	3	-3	-1	-3	0	3	4	2	-1	0	-2	-2
**12**	-2	0	-1	0	1	2	1	-1	-3	-3	2	-1	-4	0	-2	0	2	1	4	1	-1	-4	0	-3	-2	0	0	2	3	1	-2	2	3	3	-1	-2	1	4	-1
**13**	1	1	0	-2	1	0	0	-2	0	0	2	-4	-4	3	-1	1	1	0	2	2	-1	1	-1	-3	-3	3	3	2	-2	-1	-2	4	0	4	-1	-3	-1	2	-2
**14**	0	1	-2	0	2	4	-1	-3	-3	-1	-4	3	-3	0	0	0	-1	4	2	2	2	-2	1	1	-1	-2	1	3	1	-1	-4	2	-2	3	0	-1	-2	0	1
**15**	-1	2	4	2	-1	0	3	-1	0	-2	4	-1	-2	2	-2	-3	1	-1	0	1	1	-3	0	-4	-2	-1	0	0	0	2	-2	3	1	2	1	-4	1	3	-3
**16**	-1	1	0	2	0	2	3	-2	-2	-3	2	-2	-4	1	2	-3	0	-4	3	2	-1	-1	4	-1	-1	1	4	0	1	0	-2	1	1	3	0	-1	-2	0	-3
**17**	-1	1	1	3	3	1	1	-2	-1	1	2	-3	-3	0	-2	-4	0	-1	3	0	-1	2	0	-4	-2	-1	2	2	1	0	-3	4	0	4	0	-1	-2	2	-2
**18**	-1	0	-2	0	1	1	-1	-1	0	-1	3	2	-2	4	-4	-4	2	0	-2	2	0	-3	0	-3	3	1	0	1	1	-1	-2	2	2	3	1	-1	4	-3	-2
**19**	1	1	3	0	1	-2	-2	-1	-2	0	2	3	-2	0	-2	-4	2	4	-1	2	-1	1	2	-3	-1	0	-1	0	0	2	1	-1	1	4	-3	0	3	-3	-4
**20**	2	3	4	-2	0	-1	-4	0	-1	2	-1	0	2	4	1	1	3	2	1	3	1	-3	0	-3	-1	-2	-2	-2	-1	0	0	1	-1	2	-4	1	-3	-2	0
**21**	0	0	4	0	0	0	4	-2	3	-4	2	-2	-4	3	-3	1	2	1	-2	1	-1	-2	-1	-3	-2	1	0	3	0	-1	2	2	1	2	-3	-1	-1	-1	1
**22**	-1	3	0	1	0	-2	2	-1	-3	0	2	0	-2	4	-1	-1	2	0	1	4	-1	-2	1	-2	-2	-3	2	1	3	-1	-4	1	2	3	-4	0	-3	0	1
**23**	-1	1	2	4	3	2	4	-3	2	-1	0	-2	-4	3	-3	-1	-2	0	1	1	0	-2	2	-1	-3	-1	2	1	0	0	-4	3	1	0	1	-1	-2	0	-2
**24**	0	0	-4	2	1	3	-2	-3	-1	-2	-1	-1	-1	-1	-3	1	2	1	3	2	1	0	-2	-4	-2	0	2	1	4	4	-1	0	2	3	-2	1	-3	0	0
**25**	0	1	-2	2	2	3	0	2	-1	-2	2	-1	-4	4	-1	-4	-2	1	0	0	4	-2	-1	-3	1	0	1	0	2	3	-1	1	3	-1	-2	-3	0	1	-3
**26**	0	2	3	1	-1	4	2	1	4	1	2	-3	-3	3	0	-2	0	-1	-1	0	0	0	2	0	-3	-1	-2	1	1	-2	-2	2	-1	1	3	-4	-1	-4	-2
**27**	-1	1	-1	0	3	4	0	-2	1	-1	-3	-1	2	2	-2	-3	1	1	4	2	0	-3	0	-4	-2	0	1	-2	1	2	-1	3	0	3	2	-2	0	-1	-4
**28**	1	2	2	3	0	1	-2	-1	0	-1	0	-4	-3	3	-1	-1	2	1	2	0	1	-3	-1	-2	-2	0	4	-1	3	2	-2	1	0	4	1	-4	0	-2	-3
**29**	2	2	3	4	3	-2	-1	-2	-4	0	-2	-2	-3	-3	-1	-3	1	2	2	1	0	0	1	0	1	-1	-1	2	-1	0	0	1	0	4	-2	-1	1	3	-4
**30**	0	1	0	1	1	4	0	-2	-2	-1	-1	0	-3	0	-1	2	2	1	2	2	-2	-4	-3	-4	0	-2	-1	4	1	2	3	0	-1	3	1	-3	3	-2	-1
**31**	1	0	2	-1	3	2	1	-4	-2	0	-1	0	0	2	-3	-1	4	0	-1	0	1	-2	-2	-4	-2	-1	-2	4	1	3	1	2	2	3	-3	1	-3	-1	0
**32**	-1	0	1	2	2	-1	1	3	-1	-1	-3	-3	-4	0	3	-3	-2	1	-1	1	3	0	2	-2	1	2	2	-1	0	-2	-2	1	4	4	0	-2	0	0	-4
**33**	1	2	2	1	0	0	2	-3	3	0	0	0	-2	1	0	-4	-2	-3	1	4	-2	-1	3	-1	0	-1	2	-1	1	-2	-1	2	3	1	4	-4	-2	-1	-3
**34**	-1	-1	3	1	1	1	-3	-2	1	2	-2	2	-3	0	-1	-4	2	0	0	3	2	1	3	-4	0	0	-1	4	0	1	-2	2	0	4	-2	-1	-1	-2	-3
